# Deciphering the Causative Role of a Novel *APC* Gene Variant in Attenuated Familial Adenomatous Polyposis Using Germline DNA-RNA Paired Testing

**DOI:** 10.3390/biomedicines14010087

**Published:** 2026-01-01

**Authors:** Giovanna Forte, Candida Fasano, Matteo Iacoviello, Valentina Grossi, Martina Lepore Signorile, Katia De Marco, Paola Sanese, Antonia Lucia Buonadonna, Andrea Manghisi, Nicoletta Maria Tutino, Vittoria Disciglio, Cristiano Simone

**Affiliations:** 1 Medical Genetics, National Institute of Gastroenterology, IRCCS “Saverio de Bellis” Research Hospital, 70013 Castellana Grotte, Italy; giovanna.forte@irccsdebellis.it (G.F.); candida.fasano@irccsdebellis.it (C.F.); matteo.iacoviello@irccsdebellis.it (M.I.); valentina.grossi@irccsdebellis.it (V.G.); martina.lepore@irccsdebellis.it (M.L.S.); katia.demarco@irccsdebellis.it (K.D.M.); paola.sanese@irccsdebellis.it (P.S.); lucia.buonadonna@irccsdebellis.it (A.L.B.); andrea.manghisi@irccsdebellis.it (A.M.); nicoletta.tutino@irccsdebellis.it (N.M.T.); 2 Medical Genetics, Department of Precision and Regenerative Medicine and Jonic Area (DiMePRe-J), University of Bari Aldo Moro, 70124 Bari, Italy

**Keywords:** Familial adenomatous polyposis (FAP), adenomatous polyposis coli (*APC*) gene, germline DNA-RNA paired testing, *APC* gene splicing variant, *APC* gene truncating variant

## Abstract

**Background/Objectives**: Familial adenomatous polyposis (FAP) is an autosomal dominant disorder caused by pathogenic variants in the adenomatous polyposis coli (*APC*) gene. Its attenuated form (AFAP) is characterized by fewer colorectal polyps and later onset of colorectal cancer. We aimed to characterize the molecular effects of a novel *APC* gene variant (NM_000038.6: c.1620_1624delinsT) identified in a patient with AFAP. **Methods**: A 56-year-old man with the AFAP phenotype underwent germline testing via a multigene NGS panel, which identified a novel *APC* gene variant (NM_000038.6: c.1620_1624delinsT). In silico analyses predicted disruption of the canonical donor splice site and a frameshift followed by the introduction of a premature stop codon. The transcriptional impact of the identified *APC* gene variant was investigated by mRNA analysis. **Results**: mRNA analysis revealed two distinct *APC* transcripts: the first transcript led to a truncated protein (p.Leu540PhefsTer8), and the second transcript lacked exon 12, resulting in an in-frame 26 amino acid deletion of APC protein (p.Ala517_Gly542del). The transcript lacking exon 12 was more abundant than the transcript with a premature stop codon, likely due to degradation through nonsense-mediated decay. **Conclusions**: The *APC* gene variant (NM_000038.6: c.1620_1624delinsT) exhibits a dual transcriptional effect, revealing its pathogenic role in AFAP. This study highlights the diagnostic value of combined DNA–RNA germline testing for improving the clinical classification of novel *APC* gene variants and their genotype–phenotype correlations in FAP.

## 1. Introduction

Colorectal cancer (CRC) is one of the most common cancers worldwide, mainly occurring sporadically [1,2]. Approximately 10% of CRC cases are associated with inherited syndromes, with familial adenomatous polyposis (FAP) and Lynch syndrome (LS), both of which are inherited in an autosomal dominant manner, accounting for approximately 1% and 2–3% of cases, respectively [3,4,5,6]. FAP is characterized by the development of multiple (classically hundreds to thousands) premalignant adenomas throughout the colon and rectum, which, if left untreated, progress to CRC [7]. Patients with FAP have an increased risk of extra-colonic manifestations involving several organs, such as desmoid tumors, hepatoblastoma, and thyroid cancer [8]. FAP is caused by germline pathogenic variants (PVs) involving the adenomatous polyposis coli (*APC*) tumor suppressor gene. The *APC* gene is located at the 5q21-q22 locus and encodes a large, multifunctional protein involved in principal cellular processes, including the regulation of Wnt signaling, cell adhesion, and cytoskeletal dynamics [9]. The phenotypic clinical manifestations and severity of FAP are variable and are associated with the specific type and location of germline PVs within the *APC* gene [10]. Depending on the number of colorectal polyps and age of onset, FAP can be classified into distinct phenotypes, including mild, intermediate, and severe, with severe and intermediate phenotypes representing the classic form of FAP [11]. The most severe clinical form of FAP is associated with loss-of-function (LOF) variants in codons 1250 and 1424 of the *APC* gene. It is characterized by the presence of hundreds to thousands of adenomatous polyps arising during adolescence and early-age-onset CRC and is termed “profuse FAP”. The milder form of FAP, linked to LOF variants in codons 1596–2051 and near the 5′ end of *APC* (upstream codon 157), or to aberrant splicing events, including skipping of exon 9 and exon 12, is characterized by fewer polyps (10–100) and a lower risk of CRC and is termed attenuated FAP (AFAP). Intermediate FAP, in which hundreds of polyps usually develop in the second/third decade of life, and CRC, which occurs at approximately 40 years of age, are linked to LOF variants in other regions of the *APC* gene [8,12]. Additionally, a novel clinical variant of FAP, termed gastric polyposis and desmoid FAP (GD-FAP), has recently been described, characterized by fewer than 50 polyps, a higher risk of developing desmoid tumors, and greater susceptibility to profuse gastric polyposis or adenomas [12]. The vast majority of *APC* PVs are nonsense or frameshift variants, which are predicted to result in premature truncation of the APC protein. Splice site genetic variants are less frequent and can result in the generation of aberrant transcripts. Rarely, in-frame exon skipping events (e.g., exon 12 or 13) have also been documented in association with the AFAP phenotype. Characterizing the molecular consequences of distinct *APC* genetic variants, including those affecting splicing, is crucial for better diagnostic and clinical surveillance strategies [13]. In this study, we identified a novel heterozygous PV within exon 12 of the *APC* gene (NM_000038.6: c.1620_1624delinsT) in a patient with the AFAP phenotype. To elucidate the molecular consequences of this variant, we performed molecular analyses at the mRNA level, demonstrating that it is associated with the generation of two distinct transcripts. The first transcript leads to the production of a truncated protein, characterized by a premature stop codon (p.Leu540PhefsTer8), likely resulting in loss of function. The second transcript lacks exon 12, resulting in an in-frame deletion of 26 amino acids (p.Ala517_Gly542del) of the APC protein. These findings highlight the clinical and molecular impact of this PV by causing the differential expression of aberrant transcript variants, which may have potentially diverse functional and clinical consequences. Our study emphasizes the importance of integrating genomic and transcriptomic analyses to comprehensively understand the pathogenic mechanisms underlying *APC* germline variants and their associated phenotypic implications.

## 2. Materials and Methods

### 2.1. Patient Recruitment

The patient underwent a thorough clinical evaluation, including a detailed review of their personal and family medical histories. A three-generation pedigree was constructed to document familial patterns of cancer and polyposis. The collected data included age at diagnosis, type of neoplasms, and results of prior instrumental follow-up, including any genetic testing previously performed at other centers. Information obtained during genetic counseling was cross-referenced with current National Comprehensive Cancer Network (NCCN) guidelines [NCCN Clinical Practice Guidelines in Oncology for Genetic/Familial High-Risk Assessment: Colorectal, Endometrial, and Gastric. Version: 1.2025—13 June 2025. Available online at https://www.nccn.org; accessed on 10 September 2025] to determine the appropriateness of genetic testing. Molecular testing in this study was based on routine clinical diagnostic assessments performed at our institute. Written informed consent to perform genetic testing and further studies was obtained from the patient using a form approved by the competent ethics committee, in line with the principles of the Declaration of Helsinki and any other applicable local ethical and legal requirements (protocol code N° 170—date of approval 31 October 2016).

### 2.2. Germline Genetic Analysis

Genomic DNA was extracted from peripheral blood using the MagCore^®^ Genomic DNA Whole Blood Kit (Amerigo Scientific, New York, NY, USA) in accordance with the manufacturer’s instructions. A DNA sample (10 ng) was subsequently sequenced using a targeted next-generation sequencing (NGS) multigene panel (Ion AmpliSeq™ BRCA Reflex—Hereditary Cancer Research Panel) (Thermo Fisher Scientific, Waltham, MA, USA) as previously described [14]. This panel analyzed the entire coding regions of 25 genes associated with hereditary cancer, including *APC*, *ATM*, *BARD1*, *BMPR1A*, *BRIP1*, *CDH1*, *CDK4*, *CDKN2A*, *CHEK2*, *EPCAM*, *MLH1*, *MRE11A*, *MSH2*, *MSH6*, *MUTYH*, *NBN*, *PALB2*, *PMS2*, *PTEN*, *RAD50*, *RAD51C*, *RAD51D*, *SMAD4*, *STK11*, and *TP53* [14]. Gene-specific guidelines established by the Clinical Genome Consortium (ClinGen; https://clinicalgenome.org/; accessed on 10 September 2025) were used to perform germline variant classification [15]. The global population frequency of the identified genetic variants was retrieved from the gnomAD v4.1 dataset (https://gnomad.broadinstitute.org/news/2024-04-gnomad-v4-1/; accessed on 10 September 2025) [16]. The genetic variant likely responsible for the clinical phenotype of the index case was validated using Sanger sequencing. Sanger sequencing was performed using the BigDye™ Terminator Cycle Sequencing Kit (Thermo Fisher Scientific, Waltham, MA, USA), and each sample was analyzed with the SeqStudio™ Genetic Analyzer (Thermo Fisher Scientific, Waltham, MA, USA).

### 2.3. APC Gene Variant In Silico Prediction

In silico splice prediction of the *APC* gene variant (NM_000038.5: c.1620_1624delinsT) was performed using Alamut Visual Plus v1.12 (Sophia Genetics SAS, Bidart, France), which is a licensed software package integrating four splice site prediction algorithms: Splice Site Finder (SSF), MaxEntScan (MES), Splice Site Prediction by Neural Network (NNS), and Gene Splicer (GS) [17]. In silico splice prediction analysis was performed by using the default thresholds of each splice site prediction algorithm. A variation greater than 10% in at least two algorithms was deemed to affect the splicing process. Additionally, the in silico analysis of the functional effect of the small deletion and insertion affecting the coding region of *APC* was performed using the Ensembl Variant Effect Predictor (VEP), an open-source tool (https://www.ensembl.org/Tools/VEP, release 115, accessed on 10 September 2025) integrated into the Ensembl database that predicts the molecular consequences of genetic variants using the Ensembl/GENCODE or RefSeq gene sets [18].

### 2.4. Reverse-Transcription PCR (RT-PCR) and mRNA Analysis

The blood of the index case and control was collected in PAXgene Blood RNA Tubes (Qiagen, Hilden, Germany). Total RNA was extracted from the peripheral blood of the index patient using the QIAamp RNA Blood Mini Kit (Qiagen, Hilden, Germany) in accordance with the manufacturer’s instructions. Control RNA was also extracted from another individual who did not carry the *APC* PVs. One microgram of the index patient’s and control’s RNA was reverse-transcribed to cDNA using SuperScript VILO cDNA Synthesis Kit (Thermo Fisher Scientific, Waltham, MA, USA) in accordance with the manufacturer’s instructions. The 5′ and 3′ flanking region of the *APC* genetic variant site (NM_000038.5: c.1620_1624delinsT), spanning exons 10–14 of *APC*, was amplified using the primers and experimental conditions previously described [19]. PCR products were then checked by gel electrophoresis and bidirectionally sequenced using the standard protocol of the BigDye Terminator Cycle Sequencing Kit (Thermo Fisher Scientific, Waltham, MA, USA) and the SeqStudio™ Genetic Analyzer (Thermo Fisher Scientific, Waltham, MA, USA).

### 2.5. Analysis of APC mRNA Transcript Variants by Quantitative Reverse-Transcription PCR (RT-qPCR) and Droplet Digital PCR (ddPCR)

Quantitative reverse transcription PCR (RT-qPCR) was performed using a QuantStudio 3 Real-Time PCR System (Thermo Fisher Scientific, Waltham, MA, USA). Reaction mixtures for patients and controls cDNA were prepared using SYBR Green Master Mix (Thermo Fisher Scientific, Waltham, MA, USA) in line with the manufacturer’s instructions. Primers were designed to target the *APC* mRNA transcript variants identified through RT-PCR sequencing analysis, comprising the *APC* normal transcript, the *APC* transcript with a frameshift followed by a premature stop codon, and the *APC* transcript lacking exon 12. The primer sequences are listed in Table S1. The amplification protocol consisted of an initial denaturation at 95 °C for 10 min, followed by 40 cycles of denaturation at 95 °C for 15 s and annealing/extension at 60 °C for 60 s. All reactions were performed in triplicate, and the mean cycle threshold (Ct) values were used for subsequent analysis. The Ct values were first normalized to the housekeeping gene by subtracting the Ct values of the housekeeping gene (β-actin) from the Ct values of the target normal and aberrant *APC* transcripts (delta Ct–DCt) in the normal control and index case. Subsequently, the DCt values were transformed using the 2^−DCt^ formula to obtain relative expression quantification [20,21]. In addition to RT-qPCR, we enhanced our analysis by examining mRNA transcript variants distributions through droplet digital PCR (ddPCR). We prepared the reactions using ddPCR EvaGreen Supermix (Bio-Rad Laboratories, Hercules, CA, USA) along with transcript variants specific primers at a concentration of 200 nM, in accordance with the manufacturer’s guidelines. Each 20 μL reaction mix was converted into droplets using the Droplet Generator (Bio-Rad Laboratories, Hercules, CA, USA). The droplets were subsequently transferred to a 96-well plate, sealed, and cycled in a Thermocycler (Bio-Rad Laboratories, Hercules, CA, USA) following a structured protocol: a 5 min denaturation step at 95 °C, followed by 40 cycles at 95 °C for 30 s and 60 °C for 1 min, a post-cycling step for signal stabilization at 4 °C for 5 min and 95 °C for 5 min, and a final infinite hold at 4 °C. The plate was then analyzed using the QX200 Reader (Bio-Rad Laboratories, Hercules, CA, USA), and the resulting data were processed with Bio-Rad QX Manager Software Standard Edition Version 1.2 (Bio-Rad Laboratories, Hercules, CA, USA). This comprehensive approach enables a thorough understanding of the expression levels of normal and aberrant *APC* transcripts.

## 3. Results

### 3.1. Clinical and Molecular Findings

**Figure 1:**
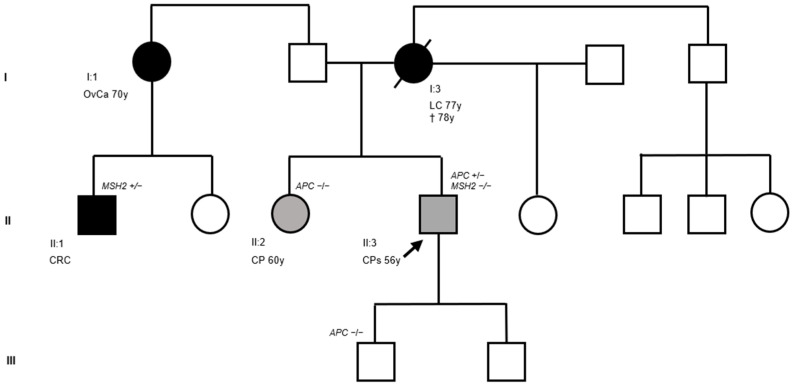
Pedigree of the family included in this study. Squares indicate men and circles women. The arrow indicates the index case. Unfilled symbols indicate unaffected individuals. Slashed symbols denote dead individuals. Black-filled symbols represent individuals with cancer. Gray-filled symbols denote individuals with colorectal polyps (CPs), and unfilled symbols denote individuals without cancer. Roman numerals (I–III) denote the generation to which each individual belongs. Arabic numerals identify individuals according to their position in the pedigree, numbered consecutively from left to right starting at 1. The following clinical manifestations are noted below each filled symbol: (CRC = colorectal cancer; LC = lung cancer; OvCa = ovarian cancer; CP = colon polyp; CPs = colon polyps), age at diagnosis (y = years), and age of death (†). The presence and the absence of heterozygous pathogenic variants (PVs) are also shown (*APC* +/− = presence of heterozygous PV; *MSH2* +/− = presence of heterozygous PV; *APC* −/− = absence of heterozygous PV).

We report the case of a 56-year-old man with an attenuated colonic polyposis phenotype in the context of a family history of LS. The family pedigree is illustrated in Figure 1.

The index case (Figure 1, II:3) was a 56-year-old male who was referred to the gastroenterology unit of our institute. He was the second-born of three siblings: he had an older sister with whom he shared both parents and a younger half-sister with whom he shared only his mother. His father and both sisters were in good health at the date of genetic counseling. His mother developed lung carcinoma at 77 years of age and died at 78 years of age. Neoplastic disease was also present on the paternal side. One of the patient’s paternal aunts was diagnosed with ovarian carcinoma at 70 years of age, and her son—the patient’s cousin—developed synchronous CRC. Genetic testing in this cousin had previously identified a germline PV in the MutS Homolog 2 (*MSH2*) gene (NM_000251.2: c.811_814del; p.Ser271Argfs*2), leading to a molecular diagnosis of LS. Unfortunately, both the patient’s aunt and father declined segregation analysis, and no information is available regarding any prior colonoscopy examinations in these relatives. Despite the absence of gastrointestinal symptoms, the patient underwent a surveillance colonoscopy, which revealed numerous (<100) colonic polyps. He subsequently underwent prophylactic colectomy. Histopathological examination of the surgical specimen demonstrated multiple tubular and tubular-villous adenomas, some with high-grade dysplasia. The older sister also underwent a surveillance colonoscopy, which revealed only a single polyp. Given the patient’s personal history, genetic analysis was performed.

### 3.2. Identification of a Novel APC Germline Variant (NM_000038.6: c.1620_1624delinsT)

**Figure 2:**
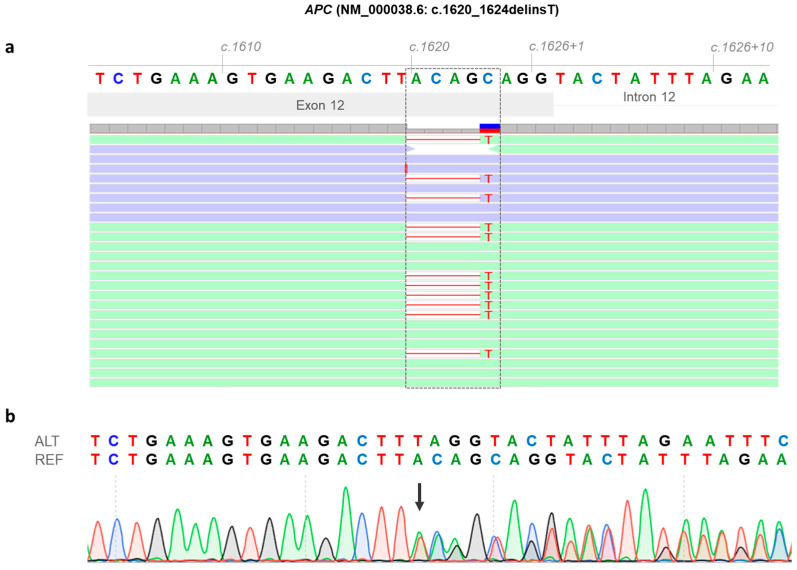
Identification of the *APC* (NM_000038.6: c.1620_1624delinsT) germline variant in the index case. (**a**) Next-generation sequencing (NGS) results showing the *APC* gene variant (NM_000038.6: c.1620_1624delinsT) in the index case. Read alignment highlights the end of exon 12 and the beginning of intron 12 of the *APC* gene DNA sequence. The gray dashed box shows the *APC* variant, consisting of a five-nucleotide deletion and insertion of the T nucleotide (NM_000038.6: c.1620_1624delinsT). (**b**) Sanger sequencing electropherogram of genomic DNA from the index case, confirming the *APC* gene variant (NM_000038.6: c.1620_1624delinsT), is shown by a black arrow. The *APC* DNA reference sequence (REF) and the *APC* DNA altered sequence (ALT) are indicated at the top of the *APC* gene electropherogram sequence.

Next-generation sequencing (NGS) of the index case revealed a heterozygous small insertion and deletion involving the *APC* gene (NM_000038.6: c.1620_1624delinsT). Moreover, NGS analysis excluded the presence of the *MSH2* (NM_000251.2: c.811_814del; p.Ser271Argfs*2) gene variant in the DNA extracted from lymphocytes of the index case. The *APC* gene variant (NM_000038.6: c.1620_1624delinsT) affects exon 12 and was confirmed by Sanger sequencing in genomic DNA from the index case (Figure 2a,b). This variant was not found in the index case’s sister (Figure 1, II:2). The index patient’s mother (Figure 1, I:3) had already died at the time of this study; therefore, her *APC* mutational status could not be assessed. However, since apparently the index case’s father and mother did not have colonic polyposis nor FAP-associated malignancies, it is reasonable to hypothesize that the identified germline *APC* variant (NM_000038.6: c.1620_1624delinsT) occurred de novo in the index case.

### 3.3. Molecular Effects of the APC Gene Variant (NM_000038.6: c.1620_1624delinsT)

**Figure 3:**
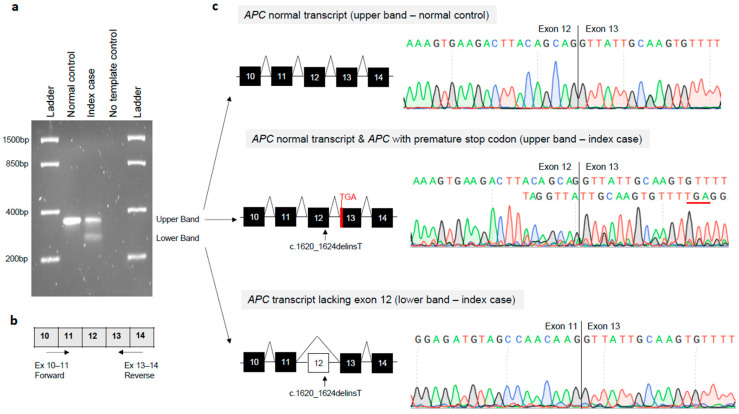
Molecular effects of the *APC* germline variant (NM_000038.6: c.1620_1624delinsT) on the *APC* transcript processing. (**a**) Agarose gel electrophoresis of reverse transcription (RT)-PCR products from the index case and a normal control. The upper and lower cDNA bands were excised from the gel and analyzed through Sanger sequencing. (**b**) Diagram showing the localization of the primers (indicated as arrows) used for RT-PCR experiments. (**c**) Sanger sequencing results showing (i) the *APC* normal transcript in the upper band of the normal control individual; (ii) the *APC* normal transcript and the *APC* with premature stop codon in the upper band of the index case; and (iii) the *APC* lacking exon 12 in the lower band of the index case.

The *APC* gene variant (NM_000038.6: c.1620_1624delinsT) was located two base pairs upstream of the exon 12–intron 12 junction, adjacent to the splice donor site of the exon 12, and consists of the deletion of 5 nucleotides (ACAGC) and the insertion of the T nucleotide. This variant was found to be rare, as it was not listed in the global population databases of gnomAD v4.1 (https://gnomad.broadinstitute.org/news/2024-04-gnomad-v4-1/; accessed on 10 September 2025). Moreover, this variant has never been reported in major disease-associated databases [Human Gene Mutation Database (HGMD): https://www.hgmd.cf.ac.uk/ac/index.php, accessed on 10 September 2025 and Clinvar: https://www.ncbi.nlm.nih.gov/clinvar/, accessed on 10 September 2025] [22,23]. In silico splicing prediction tool algorithms (SSF, MES, NNS, and GS) integrated into Alamut Visual Plus version 1.12 (Sophia Genetics SAS, Bidart, France, accessed on 10 September 2025) revealed that the *APC* gene variant (NM_000038.6: c.1620_1624delinsT) may result in a splice defect due to loss of the canonical donor site at position c.1626 of the *APC* gene (Figure S1a,b). Additionally, in silico analysis through VEP [18] (https://www.ensembl.org/Tools/VEP, release 115, accessed on 10 September 2025) revealed that the *APC* gene variant (NM_000038.6: c.1620_1624delinsT) disrupts the *APC* DNA sequence reading frame, causing a frameshift in the coding sequence at codon 540, leading to premature termination of translation at codon 547 (p.Leu540PhefsTer8) (Figure S2a,b). To investigate the molecular effect of the identified genetic variant on *APC* transcript processing, total RNA was isolated from the index case and a normal control individual. The RT-PCR amplification of *APC* transcripts encompassing exons 11–13 revealed two distinct bands in the index case (upper and lower bands), in contrast to a single amplification product (upper band) observed in the control sample (Figure 3a,b).

**Figure 4:**
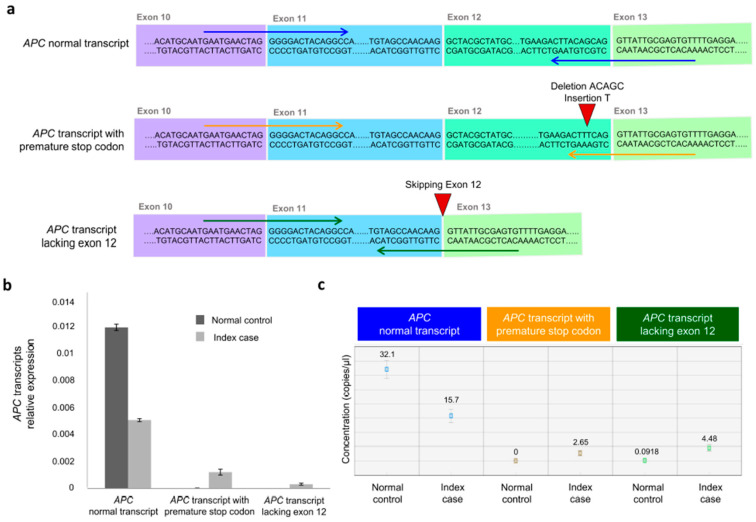
mRNA analysis of the *APC* gene variant (NM_000038.6: c.1620_1624delinsT). (**a**) Graphical representation of the *APC* mRNA transcript generated by the germline variant of the *APC* gene (NM_000038.6: c.1620_1624delinsT). The junction sequence of exons 10–11–12–13 for both the normal and aberrant *APC* transcripts (the *APC* transcript with a premature stop codon and the *APC* transcript lacking exon 12) is presented. The arrows indicate the specific RT-qPCR and ddPCR primer locations for each transcript. (**b**) RT-qPCR results are shown as histograms displaying the relative expression of the *APC* normal transcript and the *APC* aberrant transcripts in the index case and normal control. Error bars represent standard deviation. (**c**) Quantification results of the ddPCR assay (copies/μL) of *APC* mRNA expression for normal transcript and aberrant transcripts (*APC* with premature stop codon and *APC* lacking exon 12), as processed by Bio-Rad QX Manager Software Standard Edition Version 1.2 (Bio-Rad Laboratories, Hercules, CA, USA). mRNA extracted from a wild-type *APC* individual was used as a normal control. The error bars represent the maximum and minimum Poisson distribution for the 95% confidence interval generated by Bio-Rad QX Manager Software Standard Edition Version 1.2 (Bio-Rad Laboratories, Hercules, CA, USA).

Sanger sequencing of gel-purified products demonstrated that the upper band of the normal control sample corresponded to the canonical splicing pattern (exon 12–exon 13). Sanger sequencing of the upper band of the index case revealed two different *APC* transcripts, including the transcript with a canonical splicing pattern (exon 12–exon 13) and the transcript with a premature stop codon. The latter transcript showed reduced electropherogram peak intensity corresponding to the mutated allele (Figure 3c). Sanger sequencing of the lower band of the index case revealed an aberrant splicing band that skipped complete exon 12 (exon 11–exon 13) (Figure 3c). Exon 12 of the *APC* gene consists of 78 base pairs. Consequently, this aberrant splicing defect led to a 26 amino acid in-frame deletion (p.517_542del) of the APC protein. RT-qPCR and ddPCR were used to assess the abundance of normal and aberrant *APC* transcripts at the mRNA level (Figure 4).

RT-qPCR quantified all isoforms in the patient sample and controls. Quantitative analysis revealed that both aberrant *APC* transcripts (*APC* transcript with a premature stop codon and *APC* transcript lacking exon 12), which were barely detectable in the controls, were highly expressed in the index case sample. Additionally, the *APC* transcript lacking exon 12 showed a higher expression than the *APC* transcript with a premature stop codon. These findings were validated by absolute quantitative analysis using ddPCR, which confirmed that the aberrant *APC* transcripts were expressed in the index case, whereas they were detected at lower or undetectable levels in the normal control. Additionally, the expression level of the *APC* transcript lacking exon 12 was higher than that of the *APC* transcript with a premature stop codon in the index case.

## 4. Discussion

In this study, we clinically and molecularly characterized a novel heterozygous *APC* germline variant, consisting of a deletion of five nucleotides and an insertion of one nucleotide at the 5′ end of exon 12 (NM_000038.6: c.1620_1624delinsT). This *APC* germline variant was identified in a patient with the attenuated form of FAP. Sequencing analysis performed on the mRNA extracted from lymphocytes of the index case revealed that this variant has a dual molecular effect on transcription, which includes a frameshift followed by premature termination (p.Leu540PhefsTer8) and a splicing defect in which exon 12 of the *APC* gene is skipped, generating an in-frame deletion of 26 amino acids (p.Ala517_Gly542del) in the APC protein. According to the ClinGen-InSiGHT APC-specific ACMG/AMP variant classification criteria guidelines (https://cspec.genome.network/cspec/ui/svi/doc/GN089, accessed on 12 December 2025), the *APC* germline variant (NM_000038.6: c.1620_1624delinsT) can be classified as pathogenic (PVS1_Strong, PS2_Moderate, PS3_Very Strong, PM2_Supporting, PM6_Supporting, PP3_Supporting). The *APC* gene encodes a multifunctional protein (2843 amino acids) containing multiple functional regions that have been implicated in several cellular functions [24]. The APC N-terminal region (aa 1–1000) includes the oligomerization domain and armadillo repeats (ARMs) [25,26,27]. The APC middle region (aa ~1000–2100) is implicated in the binding sites for the Wnt signaling complex (axin and β-catenin) and consists of a conserved sequence of about 20 amino acids containing a Ser-Ala-Met-Pro motif (SAMP repeats) and seven 20 amino acid β-catenin-binding repeats [28]. The C-terminal region (aa ~2100–2843) includes a basic microtubule-binding domain and sites for EB1 and DLG protein bindings [29]. Through these domains, APC regulates cytoskeletal organization and plays a central role in Wnt signaling [30]. The *APC* germline variant (NM_000038.6: c.1620_1624delinsT) that generates a transcript with a premature stop codon is predicted to translate to a truncated protein (p.Leu540PhefsTer8) with loss of the principal functional domain of APC involved in β-catenin regulation. The same *APC* gene variant that generates a transcript with the loss of exon 12 (p.Ala517_Gly542del) leads to an almost complete loss of the ARM2 (aa 505–547) domain. In our recent study, we reported that the *APC* exon 12 or exon 13 splicing variant, which leads to an in-frame protein and removes ARMs motifs, may result in an attenuated form of FAP [19]. Although the precise effects of the ARM2 deletion are still unclear, it is tempting to hypothesize that this alteration may induce conformational changes in protein structure and dynamics, resulting in impaired physical interaction between APC and its binding partners, including β-catenin [19]. In contrast, truncating *APC* germline variants located in the ARM2 (aa 505–547) and/or ARM3 (aa 548–591) motifs and disrupting all downstream APC regions leads to a complete lack of regulation of the β-catenin protein, causing the classic clinical variant of FAP [19]. Based on the clinical phenotype (fewer than 100 colorectal polyps at mid-adulthood, absence of CRC , and absence of extra-colonic manifestations), the index case carrying the newly identified *APC* gene variant was classified as manifesting AFAP. Recent studies have demonstrated the utility of incorporating RNA sequencing into germline genetic testing for the diagnosis of hereditary cancer syndromes, including FAP, to identify and improve the classification of disease-causing variants [31,32]. Molecular analysis of the mRNA sample from the index case revealed the detection of two *APC* aberrant transcripts. Quantitative and semiquantitative analyses showed that the most abundant transcript was represented by the *APC* mRNA lacking exon 12. The finding that the *APC* mRNA transcript with a frameshift mutation followed by a premature stop codon was less abundant than the splicing transcript suggests that its lower expression was likely due to nonsense-mediated mRNA decay (NMD). This conserved surveillance mechanism degrades mRNAs containing premature termination codons, inducing haploinsufficiency as the most predictable pathogenic mechanism for heterozygous germline PVs [33]. Taken together, the clinical and molecular findings suggest that the *APC* gene variant (NM_000038.6: c.1620_1624delinsT) is disease-causing and can be associated with the AFAP phenotype of the index case. The attenuated clinical phenotype observed in the patient may be explained by the expression balance between the two aberrant transcripts, with the exon 12 skipped transcript being more abundant than the prematurely terminated one, which likely undergoes degradation through NMD. Despite substantial molecular characterization evidence, a limitation of our study is the lack of molecular validation of the functional effect of the *APC* variant (NM_000038.6: c.1620_1624delinsT) in the colon tissue (normal and adenomatous polyps) of the index case. Additionally, further studies investigating various aspects of mRNA metabolism, including processing, export, stability, and translational control, are needed to further elucidate the impact of this *APC* gene variant on disease pathogenesis and FAP clinical manifestations. Another limitation of the present study is that it investigated only one patient for the clinical and molecular characterization of the identified *APC* gene variant (NM_000038.6: c.1620_1624delinsT), which may reduce the generalizability of our findings. Further studies involving individuals carrying this *APC* gene variant would be helpful to support the validation of its potential clinical relevance in FAP.

## 5. Conclusions

This study demonstrated the utility of integrating blood-based RNA analysis with DNA sequencing. The DNA-RNA paired analysis provides an opportunity to more accurately characterize the molecular effect of the identified *APC* germline variant, define the underlying mechanism of pathogenesis, and support genotype–phenotype correlations in clinical manifestations of FAP. Altogether, the clinical and molecular findings of this study support the pathogenic role of a novel *APC* gene variant (NM_000038.6: c.1620_1624delinsT) and its association with the attenuated form of FAP. The association of DNA-RNA paired testing with clinical phenotyping is crucial for personalizing surveillance and management strategies in patients with FAP syndrome.

## Data Availability

Raw data of in silico prediction analysis are openly available in FigShare at https://doi.org/10.6084/m9.figshare.30328543.v1. Patient’s genetic raw data for this study are not publicly available due to confidentiality concerns and the sensitive nature of the data.

## Supplementary Materials

The following supporting information can be downloaded at: https://www.mdpi.com/article/10.3390/biomedicines14010087/s1, Figure S1. Screenshot from the Alamut Visual Plus software v1.12 (Sophia Genetics SAS, Bidart, France). Splicing effect window around the *APC* gene variant (NM_000038.6: c.1620_1624delinsT). Figure S2. Predicted effect of the *APC* gene variant (NM_000038.6: c.1620_1624delinsT) leading to a truncated protein. Table S1. Primers used for quantitative reverse transcription PCR (RT-qPCR) and droplet digital PCR (ddPCR) of *APC* gene transcripts to evaluate the molecular effect of the *APC* gene variant (NM_000038.5: c.1620_1624delinsT).

## Abbreviations

The following abbreviations are used in this manuscript:

APC	Adenomatous polyposis coli
AFAP	Attenuated familial adenomatous polyposis
ALT	DNA altered sequence
ARMs	Armadillo repeats
ClinGen	Clinical Genome Consortium
CRC	Colorectal cancer
Ct	Cycle threshold
DCt	Delta Ct
ddPCR	Droplet digital PCR
FAP	Familial adenomatous polyposis
GD-FAP	Gastric polyposis and desmoid FAP
GS	Gene Splicer
LOF	Loss-of-function
LS	Lynch syndrome
MES	MaxEntScan
NCCN	National Comprehensive Cancer Network
NGS	Next-generation sequencing
NMD	nonsense-mediated mRNA decay
NNS	Splice Site Prediction by Neural Network
OvCa	Ovarian cancer
PVs	Pathogenic variants
REF	DNA reference sequence
RT-qPCR	Quantitative reverse transcription PCR
RT-PCR	Reverse transcription PCR
SAMP	Ser-Ala-Met-Pro motif
SSF	Splice Site Finder
VEP	Variant Effect Predictor
