# Supplementary material for: Deciphering the Causative Role of a Novel APC Gene Variant in Attenuated Familial Adenomatous Polyposis Using Germline DNA-RNA Paired Testing
Source: Biomedicines. 2026 Jan 1;14(1):87. doi: 10.3390/biomedicines14010087 (PMC12838133; doi:10.3390/biomedicines14010087)
Supplement: Supplementary file 1 [file biomedicines-14-00087-s001.zip › Figure S1.pdf]

a

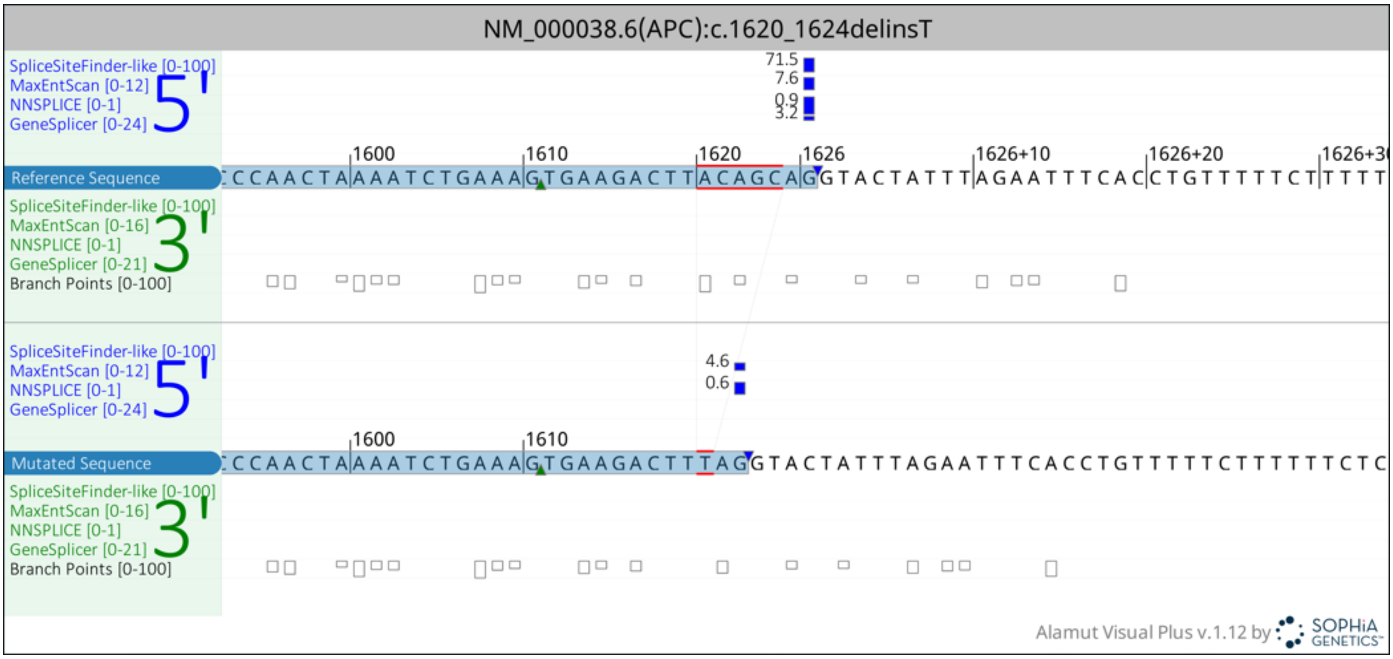

b

	**SSF [0–100]**	**MaxEnt [0–12]**	**NNSPLICE [0–1]**	**GeneSplicer [0–24]**
Threshold	≥ 70	≥ 0	≥ 0.4	≥ 0
Exon 12 – c.1626 ↘	**71.49 => –**	**7.64 => 4.64 (-39.2%)**	**0.90=> 0.62(-31.0%)**	**3.17 => –**

Figure S1. Screenshot from the Alamut Visual Plus software v1.12 (Sophia Genetics SAS, Bidart, France). Splicing effect window around the *APC* gene variant (NM_000038.6: c.1620_1624delinsT).
(a) The top box represents the DNA reference sequence of the *APC* gene; the bottom box represents the *APC* mutated sequence with the *APC* gene variant (NM_000038.6: c.1620_1624delinsT). The dark blue bars represent the predicted splice donor site.
(b) The diagram reveals the abolition of the canonical splice donor site at position c.1626. All four tools predicted that the identified variant abolishes the canonical splice donor site.
