# Supplementary material for: Deciphering the Causative Role of a Novel APC Gene Variant in Attenuated Familial Adenomatous Polyposis Using Germline DNA-RNA Paired Testing
Source: Biomedicines. 2026 Jan 1;14(1):87. doi: 10.3390/biomedicines14010087 (PMC12838133; doi:10.3390/biomedicines14010087)
Supplement: Supplementary file 1 [file biomedicines-14-00087-s001.zip › Figure S2.pdf]

a

Gene	Ensembl Gene ID (Ensembl Transcript ID)	HGSV coding sequence name	HGSV protein sequence name
APC	ENSG00000134982 (ENST00000257430.9)	ENST00000257430.9: c.1620_1624delinsT	ENSP00000257430.4: p.Leu540PhefsTer8

b

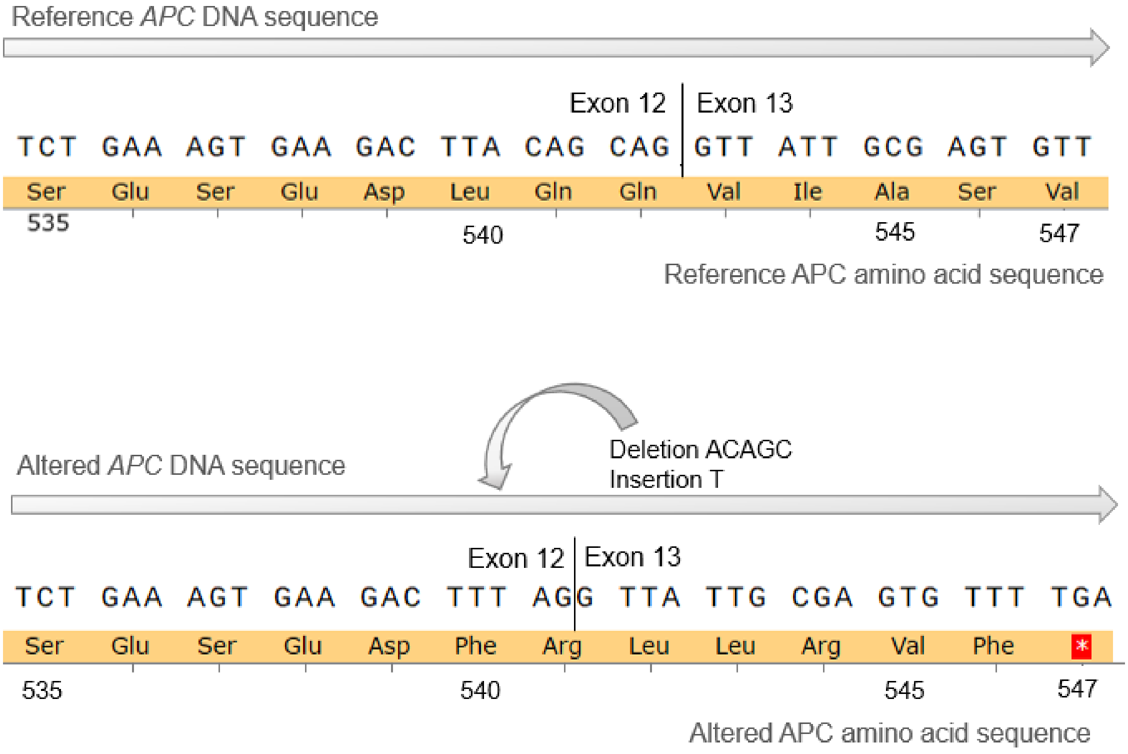

Figure S2. Predicted effect of the *APC* gene variant (NM_000038.6: c.1620_1624delinsT) leading to a truncated protein. **(a)** In silico analysis results of the *APC* gene variant (NM_000038.6: c.1620_1624delinsT) obtained using the Variant Effect Predictor (VEP) tool; **(b)** Graphical representation of the predicted effect of the *APC* gene variant (NM_000038.6: c.1620_1624delinsT). The top box represents the DNA reference sequence of the *APC* gene, which encodes a normal APC protein (reference APC amino acid sequence). The bottom box represents the DNA-altered sequence of the APC gene (NM_000038.6: c.1620_1624delinsT), disrupting the *APC* DNA sequence reading frame, causing a frameshift in the coding sequence at codon 540, leading to premature termination of translation at codon 547 (p.Leu540PhefsTer8).
