# Supplementary material for: Deciphering the Causative Role of a Novel APC Gene Variant in Attenuated Familial Adenomatous Polyposis Using Germline DNA-RNA Paired Testing
Source: Biomedicines. 2026 Jan 1;14(1):87. doi: 10.3390/biomedicines14010087 (PMC12838133; doi:10.3390/biomedicines14010087)
Supplement: Supplementary file 1 [file biomedicines-14-00087-s001.zip › Table S1.pdf]

Table S1. Primers used for quantitative reverse transcription PCR (RT-qPCR) and droplet digital PCR (ddPCR) of *APC* gene transcripts to evaluate the molecular effect of the *APC* gene variant (NM_000038.5: c.1620_1624delinsT)

***mRNA* transcript fragments**	**Forward Sequence (5′-3′)**	**Reverse Sequence (5′-3′)**
APC normal transcript	GAATGAACTAGGGGGACTACAGGC	AACACTCGCAATAACCTGCTGTAAG
APC transcript with premature stop codon	GAATGAACTAGGGGGACTACAGGC	CCTCAAAACACTCGCAATAACCTAAAG
APC transcript lacking the exon 12	GAATGAACTAGGGGGACTACAGGC	CACTTGCAATAACCTTGTTGGCTAC
β-actin	CTGGCACCCAGCACAATG	CCGATCCACACGGAGTACTTG
